# The Effect of Vestibulo-Ocular Reflex Deficits and Covert Saccades on Dynamic Vision in Opioid-Induced Vestibular Dysfunction

**DOI:** 10.1371/journal.pone.0110322

**Published:** 2014-10-20

**Authors:** Cecilia Ramaioli, Paolo Colagiorgio, Murat Sağlam, Fabian Heuser, Erich Schneider, Stefano Ramat, Nadine Lehnen

**Affiliations:** 1 German Center for Vertigo and Balance Disorders, Munich University Hospital, Munich, Germany; 2 Department of Electrical, Computer and Biomedical Engineering, University of Pavia, Pavia, Italy; 3 Department of Anesthesiology, Klinikum rechts der Isar, TU Munich, Munich, Germany; 4 Clinical Neurosciences, Munich University Hospital, Munich, Germany; 5 Brandenburg University of Technology, Cottbus-Senftenberg, Germany; 6 Department of Neurology, Munich University Hospital, Munich, Germany; UMR8194, France

## Abstract

Patients with bilateral vestibular dysfunction cannot fully compensate passive head rotations with eye movements, and experience disturbing oscillopsia. To compensate for the deficient vestibulo-ocular reflex (VOR), they have to rely on re-fixation saccades. Some can trigger “covert” saccades while the head still moves; others only initiate saccades afterwards. Due to their shorter latency, it has been hypothesized that covert saccades are particularly beneficial to improve dynamic visual acuity, reducing oscillopsia. Here, we investigate the combined effect of covert saccades and the VOR on clear vision, using the Head Impulse Testing Device – Functional Test (HITD-FT), which quantifies reading ability during passive high-acceleration head movements. To reversibly decrease VOR function, fourteen healthy men (median age 26 years, range 21–31) were continuously administrated the opioid remifentanil intravenously (0.15 µg/kg/min). VOR gain was assessed with the video head-impulse test, functional performance (i.e. reading) with the HITD-FT. Before opioid application, VOR and dynamic reading were intact (head-impulse gain: 0.87±0.08, mean±SD; HITD-FT rate of correct answers: 90±9%). Remifentanil induced impairment in dynamic reading (HITD-FT 26±15%) in 12/14 subjects, with transient bilateral vestibular dysfunction (head-impulse gain 0.63±0.19). HITD-FT score correlated with head-impulse gain (R = 0.63, p = 0.03) and with gain difference (before/with remifentanil, R = −0.64, p = 0.02). One subject had a non-pathological head-impulse gain (0.82±0.03) and a high HITD-FT score (92%). One subject triggered covert saccades in 60% of the head movements and could read during passive head movements (HITD-FT 93%) despite a pathological head-impulse gain (0.59±0.03) whereas none of the 12 subjects without covert saccades reached such high performance. In summary, early catch-up saccades may improve dynamic visual function. HITD-FT is an appropriate method to assess the combined gaze stabilization effect of both VOR and covert saccades (overall dynamic vision), e.g., to document performance and progress during vestibular rehabilitation.

## Introduction

Patients with bilateral vestibular dysfunction cannot produce eye movements that are fully compensatory of passive head rotations, for example during walking [1] or driving [1], [2]. This causes disturbing oscillopsia [3] even if the resulting retinal slip is small [4], [5]. Oscillopsia significantly impairs quality of life [1], [2]. To compensate for their deficient vestibulo-ocular reflex (VOR), patients have to rely on re-fixation saccades. Some can trigger shorter-latency re-fixation saccades occurring while the head is still moving (“covert” saccades, [6]) whereas others only initiate “overt” saccades after head movement end. Due to their shorter latency, it has been hypothesized that covert saccades are advantageous to stabilize gaze, reduce oscillopsia [6], [7] and improve visual performances, e.g., reading ability during head movements. To our knowledge, so far, no diagnostic test has been shown to document the effect of early saccades on visual performance during head movements. With oscillopsia being a main determinant for quality of life impairment in patients with bilateral vestibulopathy [1], such a diagnostic test would be useful. Vestibular dysfunction and the presence of covert and overt saccades can be quantified with the search-coil [8], [9] and the video [10], [11] head-impulse test [12]. These tests do not assess functionality. Although they indicate a deficit, they do not provide results directly related to visual performance. Functionality, i.e. visual perception/acuity during head movements is frequently evaluated by dynamic visual acuity tests [13]–[15]. Here, we use a recently proposed approach, the Head Impulse Testing Device – Functional Test (HITD-FT), which assesses reading performance during passive high-acceleration, head movements [16], [17].

Vestibular dysfunction was transiently introduced pharmacologically in healthy subjects by the short-acting opioid remifentanil, to investigate whether the HITD-FT was useful to assess dynamic vision, i.e., the effect of VOR and early catch-up saccades on visual perception during physiologically relevant head movements.

We had two hypotheses:

1. The HITD-FT is a useful tool to assess the combined effect of VOR and early catch-up saccades during high-acceleration, physiological head movements, and
2. Early catch-up saccades improve dynamic visual function.

## Materials and Methods

### Subjects

Fourteen healthy men aged 21–31 years (median 26 years) volunteered. They reported no history of balance or neurological disorders, and did not take any medication, in particular no opioids.

### Ethics statement

The Ethics Committee of the Technical University of Munich approved the study, which was conducted in accordance with the principles expressed in the Declaration of Helsinki. All subjects gave their written informed consent prior to participation, and were free to withdraw from the experiment at any time.

### Opioid administration

Remifentanil was administered continuously through a cubital vein at a rate of 0.15 µg/kg/min. Remifentanil was chosen because it quickly reaches a steady-state plasma level (90% after 17 minutes) and has a short half-time after stopping administration (3.7 minutes) [18]. Therefore, its effect is rapid and reversible. Standard anesthesiology monitoring was applied using ECG, non-invasive blood pressure and pulse oxymetry.

### Experimental set-up

Each subject was tested before remifentanil administration and with the drug having been infused for at least 45 minutes (“during remifentanil”). For each test, an experienced examiner standing behind the subjects performed passive, high-acceleration (2000–7000°/s^2^) and small amplitude (10–20°) head rotations to the left and right in the plane of the horizontal semicircular canals (standard head-impulses [12]) while subjects fixated a visual stimulus 2 meters straight ahead. The visual stimulus consisted of a standard Landolt ring, which had a gap measuring 1/5 of the ring diameter, and with eight possible gap positions at 45° increments [17]. Impulses were delivered with random timing and direction, to prevent anticipatory compensatory movements. For each subject, the size of the Landolt ring was 0.6 logMAR bigger than the static visual acuity determined according to Ramat et al. [16] before remifentanil infusion. The stimulus was presented on a 60×53 cm monitor with a resolution of 1280×800 pixels and a refresh rate of 75 Hz. The actual timing of the visual stimulus was documented with a photodiode taped to the monitor [17]. The visual stimulus appeared on the screen 80±2 ms (mean±SD) after head acceleration reached 300°/s^2^. Display duration was 80±8 ms. Subjects had to identify the position of the gap. They provided answers using an external computer keypad consisting of buttons for each gap position. Subjects pressed a special button if they had low confidence in their answer. The latter was introduced to further reduce the possibility of random correct answers [17].

### Data acquisition

Eye movements were recorded by video-oculography of the left eye; head movements by integrated six-degrees-of-freedom inertial sensors (EyeSeeCam system in analogy to Bartl et al. [10] but without an additional camera on a bite bar). Sampling rate was 220 Hz.

### Data analysis

Data were analyzed offline using MATLAB (MathWorks, Natick, MA) software. Eye position was computed using rotation vectors. Head angular velocity was derived from the sensors mounted on the EyeSeeCam system (in analogy to Bartl et al. [10]).

Eye velocity was filtered with a third order low-pass digital Butterworth filter with a cut-off frequency of 40 Hz. Head acceleration was computed from head velocity, which was filtered by a second order zero-phase low-pass digital Butterworth filter with 30 Hz cut-off frequency (the filtfilt.m MATLAB function was used for zero-phase filtering).

Head-impulses and saccades were automatically detected using a velocity and acceleration criterion respectively, with the possibility for manual correction. Impulses slower than 80°/s (peak velocity) were discarded. Eye movements with an acceleration higher than 2000°/s^2^ were considered as re-fixation saccades. Trials during which gaze was not on target (more than 2° of retinal error) during the 25 ms before stimulus presentation were excluded. For each trial, head and eye traces along with the visual stimulation signal were shifted together so that for every trial the visual stimulus onset was the same. Saccades were considered to happen during stimulus presentation if their peak velocity was during stimulus presentation.

On average 11±6 (mean±SD) trials were used for analysis for each side. Head- impulse gain of the VOR was determined by velocity regression [8]. Head-impulse gain was considered pathological if it was <0.7 (in analogy to [11]). Gain difference was the difference between the head-impulse gains recorded on the same subject before and during remifentanil administration. The answer was rated as correct or incorrect/no answer for each trial. HITD-FT score was calculated as the rate of correct answers from all trials of one subject in one condition (with/without remifentanil).

### Statistical analysis

Data from head-impulses to the left and right side were pooled, as there was no side difference in head-impulse gain and in HITD-FT scores between before and during remifentanil administration (repeated measures analysis of variance, ANOVA, p>0.3). Differences in head-impulse gain before and during remifentanil administration were assessed using an ANOVA with a significance level of 0.05 (data were normally distributed, verified by Kolmogorov-Smirnov Test). HITD-FT scores during remifentanil administration having values of 1.5 interquartile range (IQR) higher than the third quartile and 1.5 IQR lower than the first quartile were considered outliers. Outlier analysis using two standard deviation ranges led to the same outliers. The same outlier analysis was performed for the data before remifentanil administration.

## Results

**Figure 1:**
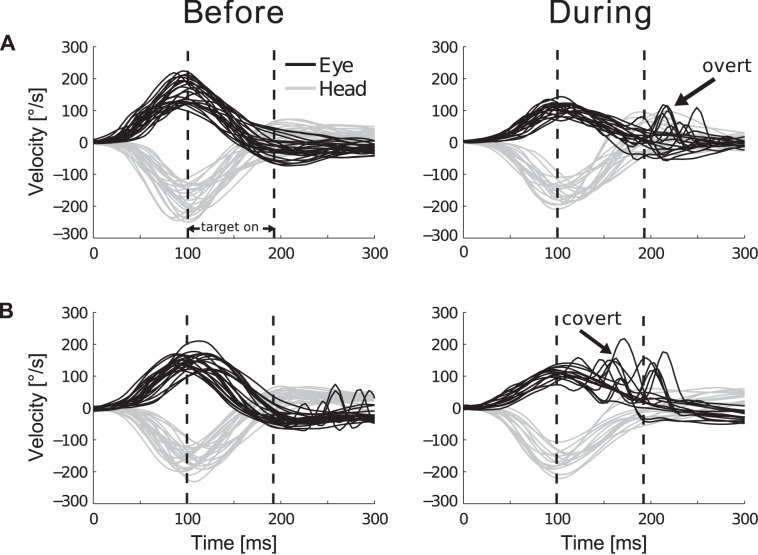
Eye and head movements during head-impulses: representative subject (A) and subject triggering covert saccades (B). Before remifentanil administration (A and B, left), the vestibulo-ocular reflex (VOR) was intact. Eye movements (black) compensated for passive head rotations (grey). Figure A, right, shows the behavior of a typical subject during remifentanil administration. The VOR was impaired (head-impulse gain 0.56±0.11). The eyes were not sufficiently compensating for the head rotation, moving in the direction of the head movement. The subject initiated re-fixation saccades. These saccades occurred after the head movement when the visual stimulus was switched off (dashed lines indicate the mean time interval during which the visual stimulus was displayed). Reading ability during head motion was impaired (HITD-FT rate of correct answers 13%). One subject (B, right) could perform catch-up saccades during stimulus presentation in 60% of the head thrusts. Reading ability was intact in spite of a decreased VOR (head-impulse gain 0.59±0.03; HITD-FT rate of correct answers 93%). Eye and head velocity traces were aligned to stimulus start.

Figure 1A shows eye and head velocity traces during head-impulse testing in one representative subject. Before remifentanil application, the VOR was intact with the eyes compensating for the head movement (head-impulse gain 0.77±0.01, mean±SD). The subject could read during the head movement (HITD-FT 100%). During remifentanil infusion, the VOR was impaired (gain 0.56±0.11), and the eyes moved in the direction of the head away from the Landolt ring. The subject performed “overt” catch-up saccades back to the ring. These catch-up saccades occurred after the visual stimulus had disappeared from the screen. Reading during the head movement was impaired (HITD-FT 13%).

**Figure 2:**
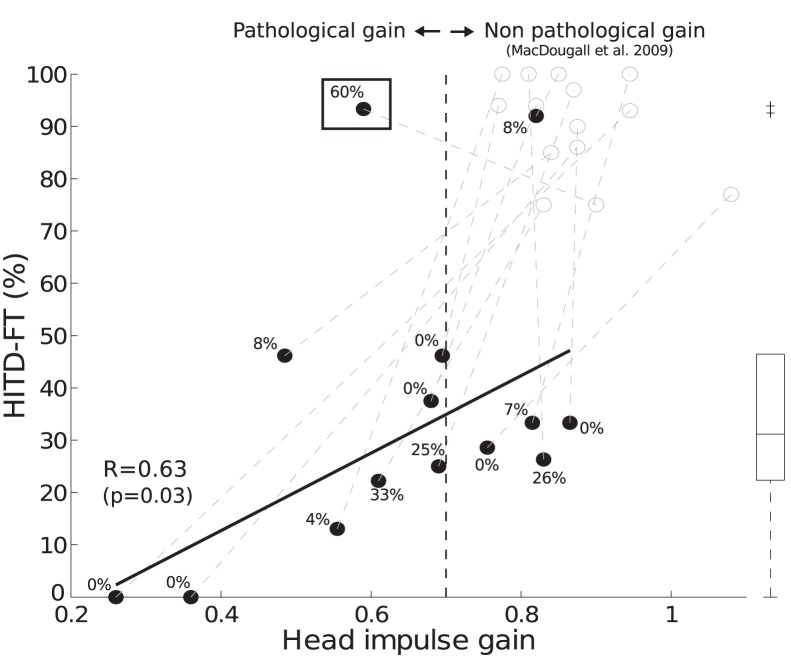
Correlation between vestibulo-ocular reflex (VOR) gain and HITD-FT scores during remifentanil administration. HITD-FT rate of correct answers and head-impulse gains are shown; each dot represents mean results from one subject, values computed during left and right head rotations were pooled. Empty dots show data for head-impulse gain and HITD-FT scores before remifentanil administration. Black dots represent data during remifentanil administration. Gray dashed lines indicate the changes within each subject. The percentage of covert saccades performed during stimulus presentation is reported for each subject. The black vertical dashed line indicates the threshold for a pathological head-impulse gain (<0.7, in analogy to [11]). For 12/14 subjects, HITD-FT scores and head-impulse gains correlated: low gain, reflecting a deficient VOR, affected the ability of reading during head movements, leading to a low HITD-FT score (solid line, R = 0.63, p = 0.03). Two subjects showed high HITD-FT scores, which fell out of the one-tailed 99% confidence interval of the HITD-FT scores from all subjects during remifentanil administration. These subjects were also the only ones being detected as outliers (see Methods); box shows interquartile range, whiskers extend to the most extreme values not considered outliers (MATLAB standard boxplot). One of these subjects showed a high HITD-FT score with a normal head-impulse gain (right upper corner). The other subject (black box, example B in Figure 1) could trigger “covert” saccades during the stimulus presentation in 60% of the head rotations. The HITD-FT was intact despite a pathological head-impulse gain.

A similar behavior was observed in 12/14 subjects. In this group, the head-impulse gain decreased from 0.87±0.09 before to 0.63±0.19 with remifentanil administration (ANOVA, p<0.001). HITD-FT score decreased as well (91±9% before and 26±15% during remifentanil). For these subjects, head-impulse gain impairment correlated with HITD-FT performance (Figure 2; Pearson correlation, R = 0.63; p = 0.03). There was a negative correlation between gain difference before and with remifentanil and HITD-FT score (Pearson correlation, R = −0.64, p = 0.02).

Two subjects fell above the 99% confidence interval of the HITD-FT results from all subjects during remifentanil administration, having high HITD-FT scores. These subjects were also the only ones being detected as outliers (see Methods).

One subject had a high HITD-FT (92%) and a normal head-impulse gain (0.82±0.03) during remifentanil administration. Another subject could read well despite a pathologically decreased head-impulse gain. Figure 1B shows individual eye and head velocity traces during head-impulses in the latter subject. Before remifentanil, the VOR was intact (0.82±0.03, mean±SD, HITD-FT 75%). During remifentanil, the head-impulse gain decreased to 0.59±0.03. The subject performed covert catch-up saccades while the Landolt ring was still visible in 60% of the imposed head rotations. HITD-FT with remifentanil was 93%. All other subjects showed covert saccades in no more than 33% of trials.

## Discussion

In summary, using the fact that remifentanil acutely and reversibly decreases VOR-function, we show that the HITD-FT reflects the combined effects of VOR and catch-up saccades on dynamic reading ability, i.e., it is a measure of overall dynamic visual function.

HITD-FT correlates with head-impulse gain impairment and detects head-impulse gain changes, as demonstrated by the correlation between HITD-FT and VOR gain differences before and during remifentanil in this study, unless there are sufficient early corrective saccades.

Unlike the conventional video head-impulse test, it also shows the effect of early corrective saccades on dynamic visual performance. In contrast to the traditional dynamic visual acuity test it exploits high acceleration head rotations, which are physiologically similar to everyday head movements [19], especially during walking [20]. This makes it a good tool for assessing the effect of vestibular rehabilitation, for example, where overall physiologically relevant dynamic visual function matters.

Our data hints that short-latency, covert catch-up saccades may indeed improve dynamic visual function: the patient with 60% catch-up saccades during stimulus presentation had a HITD-FT score similar to those achieved in the healthy condition, despite a head-impulse gain impairment. In this particular subject, therefore, covert saccades appear to be very effective in functionally supplementing the deficient VOR. In contrast, 33% of early catch-up saccades were not enough to relevantly improve dynamic visual function. To our knowledge, this has not been shown so far. Due to saccadic suppression, the subjects probably could not detect the gap position during the saccadic movement, however, the “covert“ saccades helped to foveate the target, i.e., reduce retinal error at the end of the saccade, which contributed to improve the rate of correct answers during HITD-FT.

HITD-FT thus documents the effect of covert saccades on reading ability during high-acceleration, passive, head movements. The fact that frequent covert saccades only occur in one of our 14 subjects is in line with results after vestibular schwannoma resection [21]. In the acute stage of the lesion, most patients showed overt saccades. Covert saccades occurred later post-operatively. As previously speculated [6], [7] such short-latency saccades reduce retinal slippage in vestibular dysfunction and improve dynamic visual function. This is important, as oscillopsia greatly impairs quality of life in patients with bilateral vestibulopathy [1].

In conclusion, our study suggests that HITD-FT is an appropriate method to document the gaze stabilization effect of both the VOR and covert re-fixation saccades. The combination of testing dynamic vision with head movement characteristics closely related to subjects’ daily activities (e.g., walking) makes the HITD-FT also a suitable tool for assessing overall dynamic visual performance. This will help to monitor progress in vestibular rehabilitation and to understand how to train patients to trigger early catch-up saccades to decrease oscillopsia and to design vestibular rehabilitation programs accordingly.
